# Mask-Related Glasses Fogging: A Predisposing Mechanism of Falls during the COVID-19 Pandemic

**DOI:** 10.1155/2021/5600216

**Published:** 2021-08-08

**Authors:** John F. Dankert, Mandeep S. Virk

**Affiliations:** Department of Orthopedic Surgery, NYU Langone Health, New York, New York, USA

## Abstract

Fogging is a relatively infrequent, yet annoying, issue encountered by individuals who wear glasses. With the arrival of COVID-19, glasses fogging is more common due to the ubiquitous use of face masks. Individuals are stuck wrestling between leaving their mask off or trying to navigate their day-to-day lives with fogged glasses and risk falling. We report a case of an olecranon fracture sustained due to reduced visibility secondary to mask-related fogging during the COVID-19 pandemic. The recommendations included here will provide health care providers with the necessary information to educate patients regarding prevention of mask-related glass fogging.

## 1. Introduction

Orthopedic surgeons have encountered unique challenges in providing patient care during the COVID-19 pandemic. One of the immediate threats from the virus was its impact on patients undergoing both emergent and elective orthopedic surgery. Previous work demonstrated that an active symptomatic COVID-19 infection predicted poor outcomes for patients with a traumatic musculoskeletal injury [1]. Indeed, infection with the virus was found to increase mortality and complication rates for patients admitted with a hip fracture [2]. Communities have focused on implementing societal changes to limit the impact of COVID-19.

Hand hygiene, social distancing, and masks have all become a recommended part of daily routines to reduce the spread of COVID-19. However, the near ubiquitous use of masks has left many elderly and visually impaired patients who use prescription glasses struggling with glasses fogging. Mask-related fogging can result in a fall via mechanisms including tripping, slipping, and misjudging step depth. Over 3 million geriatric patients are brought to an emergency department in the United States every year for a fall-related injury [3]. One out of every 5 falls has been reported to lead to a musculoskeletal injury in this population. During the COVID-19 pandemic, we have cared for multiple patients who have attributed their fall to impaired sight after their glasses became fogged. Off the shelf masks, commonly used in the community setting, are notorious for glasses fogging. This issue, combined with gait instability and poor bone quality, as often observed in a geriatric population, can contribute to serious fall-type musculoskeletal injuries requiring surgical treatment.

We present this case report to draw attention to mask-related glasses fogging as a mechanism for falling during the COVID-19 pandemic. This report provides information for health care professionals to apply in everyday practice with the goal of educating patients regarding the risk and prevention of falls with mask-related glasses fogging.

## 2. Case Presentation

**Figure 1:**
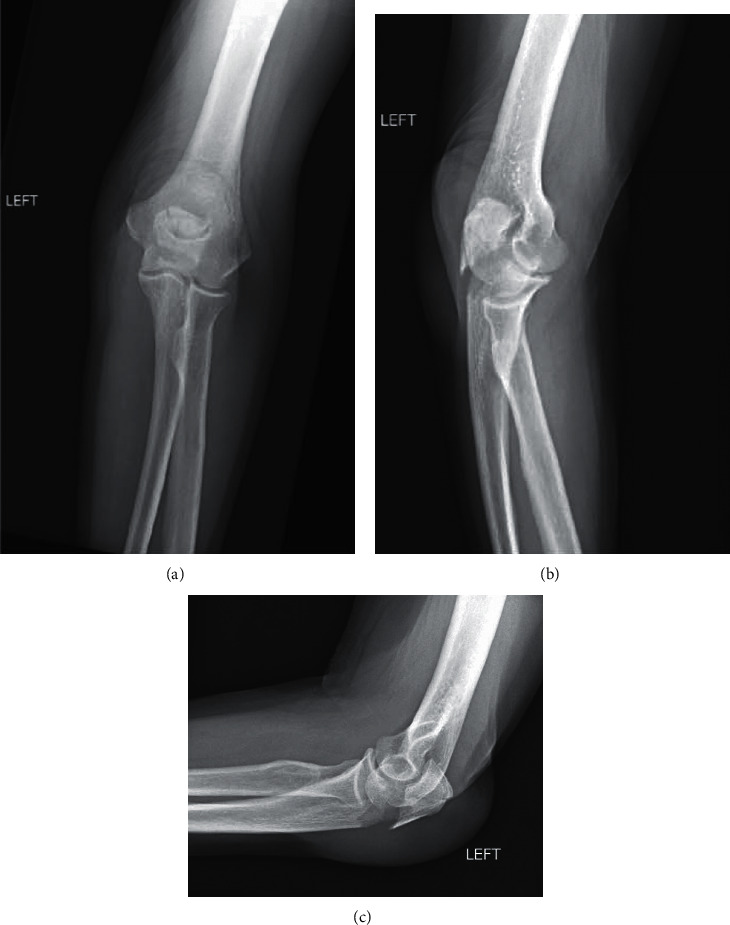
Preoperative AP (a), oblique (b), and lateral (c) radiographs demonstrating a displaced and comminuted left olecranon fracture.

**Figure 2:**
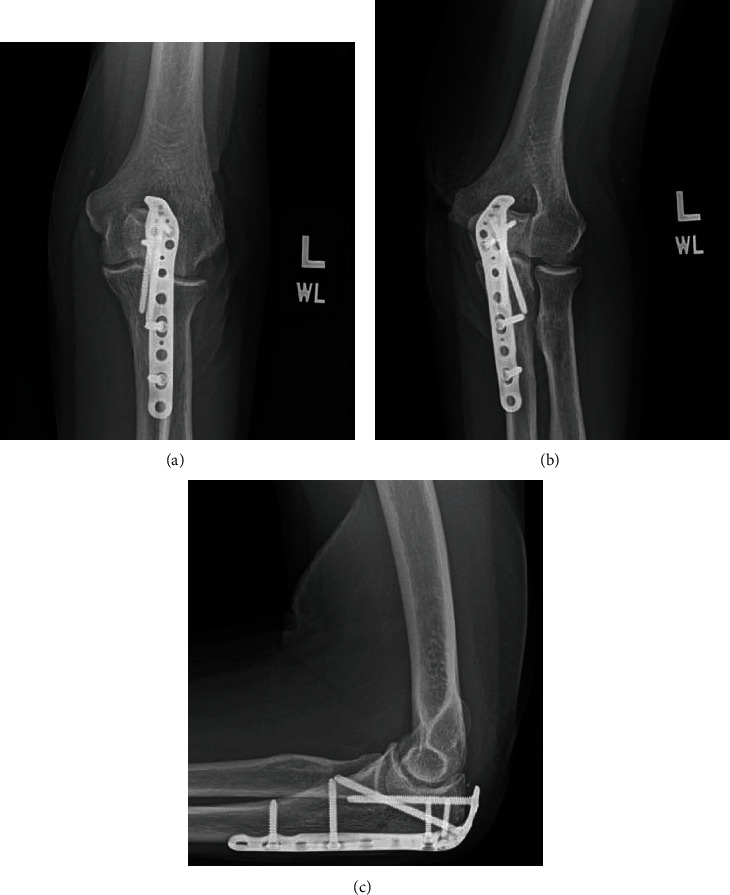
AP (a), oblique (b), and lateral (c) radiographs of the patient's left olecranon at the 3-month follow-up visit after operative fixation.

Our patient was a 70-year-old female who presented with left elbow pain after falling while walking outside. She reported that her glasses were fogged due to an ill-fitting mask causing her to misjudge her step on a raised section of the sidewalk. She landed on the point of her left elbow and complained of pain and swelling over the impact site. On physical examination, she was tender to palpation over her left olecranon with limited elbow range of motion secondary to pain. Orthogonal imaging of the left elbow demonstrated a displaced comminuted olecranon fracture (Figure 1). She was indicated for and subsequently underwent surgical repair of the olecranon fracture without any complications. At the 5-month follow-up visit, she reported only minimal pain with mild stiffness (Figure 2). She could actively flex-extend through a 10°-140° arc of motion with 80° of maximum pronation and 80° of maximum supination. Due to COVID-19 restrictions limiting her access to supervised physical therapy programs, a home exercise therapy program was prescribed to continue assisting her with reobtaining her preinjury level of function.

## 3. Discussion

Glasses fogging has been previously recognized as a problem for glasses wearers. It is considered an occupational hazard for people who wear safety glasses at work and a risk factor for eye injuries [4]. Fogging can occur from either moisture in the environment or from an individual's breath. This effect is especially apparent in cold weather because the moisture evaporates slowly from cold surfaces. When the warmer water vapor encounters a colder glass lens, it converts into a layer of droplets and results in fogging. Glasses fogging may be accentuated when wearing a mask as moisture-laden breath is directed towards the glass lenses.

We performed an extensive literature search on available solutions for preventing or minimizing glasses fogging related to mask wear. A poorly fitting mask was the primary reason for mask-related fogging in any environment [5]. Several techniques have been described to assist glasses wearers with fogging issues. These interventions are primarily based on improving the mask fit on the face or reducing the fogging phenomenon on the lenses via the application of chemical agents.

Size, shape, and accessory modifications contribute to a mask's overall fit. N95, loose-fitting surgical, and cloth masks are produced in multiple sizes to better match anatomic variations [6]. Surgical-type and cloth masks have been more popular during the COVID-19 pandemic, in part, due to the general public's lack of access to the in-demand N95 masks and the associated fit testing training required to correctly use N95 masks [7, 8]. All of these mask types often include a metal clip that may be molded over the nasal bridge. This metal clip improves the mask seal and prevents warm breath from leaking towards the glass lenses. However, the pressure from the clip can cause skin reactions after long wear times [9]. Alternatively, self-adhesive silicone pads may be placed inside a surgical-type or cloth mask to increase the seal against the patient's nose and upper maxilla. Applying an adhesive tape along the top border of the mask and adjacent skin is another trick that has been used by surgeons for years to prevent glasses fogging during procedures [5, 10]. Importantly, the repeated use of adhesive tape can cause a rash on sensitive skin and leave an area of hyperpigmentation with chronic application [11]. Alternatively, commercial masks with a built-in one-way valve to allow for the egress of breath can be helpful, but this feature increases the price of the mask [12]. Regarding the mask material, selecting a more absorbent fabric (e.g., cotton) will trap breath moisture and reduce fogging [13]. Finally, the American Academy of Ophthalmology has recommended sliding glasses down over the mask to pinch the upper part of the mask with the glasses nose pads [14]. The best method will likely depend on trial-and-error and patient preference.

Both professional and “at-home” chemical coatings are available to minimize glasses fogging. Dish soap or soap solutions, if not thoroughly washed from the lenses, can form a thin layer of antifogging coating [5]. However, too much soap residue can itself impede vision, and its antifog effect is not long-lasting. Antifog solutions are also commercially available, but these sprays and gels again require repeat application and may be expensive [15]. Finally, permanent antifog coatings are an option for select types of lenses [5]. Consultation with an optometrist or ophthalmologist will help patients in identifying an appropriate solution for their needs.

Multiple studies have investigated participants' heart rates, oxygen saturation, carbon dioxide levels, and symptoms while wearing masks. FFP2 masks covered with a surgical mask were found to be associated with increases in heart rate and nonclinically relevant drops in arterial oxygen saturation when evaluated over different wear time periods [16]. When compared to not wearing a mask, FFP2 masks were similarly found to result in significant, but not clinically relevant, increases in carbon dioxide partial pressure and decreases in oxygen saturation at different work levels [17]. When N95 and surgical masks were compared, N95 masks led to a larger increase in perioral surface temperature and higher reported sensations of humidity, heat, breathing difficulty, discomfort, and mask touching after one hour of wear [18]. Other reported symptoms included dizziness and headaches [16–18]. Assisting patients with mitigating the negative effects of mask wearing is necessary to ensure adequate protection from the COVID-19 virus.

## 4. Conclusion

Mask-related fogging has become a commonly reported menace for glasses wearers during the COVID-19 pandemic. Reduced visibility due to glasses fogging is an additional risk factor for falls especially in the elderly population where other fall predisposing risk factors like balance issues, gait disturbances, and walking aids are commonly present. The information provided in this report will allow orthopedic surgeons and other health care professionals, who may not wear glasses and not be aware of this potential issue, better assist their glasses-wearing patients with identifying solutions to reduce glasses fogging.

## Data Availability

No archived datasets were relevant to the preparation of this manuscript.
